# PVA-Cellulose Fibers Composites Impregnated with Antimicrobial Particles: The Solvent Effect

**DOI:** 10.3390/polym17182456

**Published:** 2025-09-10

**Authors:** Anca Giorgiana Grigoras, Irina Popescu, Luiza Madalina Gradinaru, Gabriela Mihalache, Florin Daniel Lipsa, Simona Luminita Nica, Vasile Cristian Grigoras

**Affiliations:** 1 ”Petru Poni” Institute of Macromolecular Chemistry, Grigore Ghica Voda Alley 41A, 700487 Iasi, Romania; 2 Integrated Centre of Environmental Science Studies in the North Eastern Region (CERNESIM), Department of Exact and Natural Sciences, Institute of Interdisciplinary Research, “Alexandru Ioan Cuza” University of Iasi, 11 Carol I, 700506 Iasi, Romania; 3 Department of Food Technologies, Faculty of Agriculture, “Ion Ionescu de la Brad” Iasi University of Life Sciences, No. 3 Mihail Sadoveanu Alley, 700490 Iasi, Romania

**Keywords:** cellulose-PVA composites, silver particles, magnetite, cryogel, wettability, antimicrobial activity

## Abstract

Nowadays, utilizing biodegradable and bio-inspired substances and combining them in innovative ways is a prerequisite for obtaining new and useful materials. In this paper, we designed and characterized eco-friendly materials as alternatives for packaging and medical applications. Thus, cellulose fibers of medical gauze or filter paper were coated with a mixed solution containing poly(vinyl alcohol) (PVA), plant-based synthesized silver particles (AgPs), and magnetite (MG). The composites and their components were studied using UV-Vis, FTIR, and Energy-Dispersive X-ray (EDX) spectroscopy, Dynamic Light Scattering (DLS), Transmission Electron Microscopy (TEM), and Scanning Electron Microscopy (SEM) to evidence the presence, size, surface charge, morphology, and chemical composition of particles in the composites, as well as particle interactions. Their proven hydrophobic and antibacterial character could recommend them for the design of antifouling medical coatings and food packaging.

## 1. Introduction

In recent years, the development of multifunctional composite materials has gained significant momentum, particularly in biomedical, environmental, and packaging applications. Among these, polymer-based composites incorporating inorganic nanoparticles have shown remarkable promise due to their enhanced mechanical, thermal, and biological properties [1]. One such promising system involves poly(vinyl alcohol) (PVA)—a water-soluble, biocompatible polymer—integrated with silver particles (AgPs) and magnetite (Fe_3_O_4_, or MG) particles, processed via cryogelation and supported on cellulose-based substrates such as gauze or paper. These composites exhibit unique features, including tunable wettability, antibacterial properties, and mechanical resilience, making them highly attractive for a wide range of practical applications.

PVA is widely used in biomedical and environmental engineering due to its non-toxic nature, biodegradability, and ability to form hydrogels through physical or chemical crosslinking. When subjected to freeze–thaw cycles, PVA undergoes physical gelation, resulting in cryogels with highly porous, interconnected network structures. This process avoids the use of potentially harmful chemical crosslinkers and creates a suitable matrix for immobilizing nanoparticles and controlling moisture absorption or diffusion [2].

Silver nanoparticles (AgNPs) are well known for their broad-spectrum antimicrobial properties, attributed to their ability to disrupt bacterial membranes and interfere with cellular metabolism. Incorporating AgNPs into polymer matrices allows for controlled release and enhanced durability of their antibacterial effects. MG nanoparticles, owing to their superparamagnetic properties, introduce additional functionality to the composite, enabling applications such as targeted drug delivery, magnetic separation, and remote actuation.

Green chemistry plays an increasingly important role in nanotechnology. In this context, plant-derived phytochemicals serve as both reducing and stabilizing agents for the eco-friendly synthesis of AgNPs. Plant extracts, rich in bioactive compounds such as polyphenols (e.g., flavonoids and phenolic acids), terpenoids, proteins, reducing sugars, and organic acids, effectively reduce silver ions (Ag^+^) to elemental silver (Ag^0^) and contribute to the nanoparticle stabilization. The specific phytochemicals and their functions vary by plant species and the extraction method used. For example, compounds such as xanthones, phloroglucinols, and naphthodianthrones have been identified as key contributors in reduction and capping roles [3,4,5]. The polyphenols exhibit strong reducing capabilities in alkaline media, facilitating the formation of AgNPs, typically evidenced by the appearance of a surface plasmon resonance (SPR) band [6].

The use of cellulose fibers, in forms such as medical gauze or paper, as a solid support enhances the mechanical integrity of the composites while providing a biocompatible and flexible substrate. The hydrophilic nature of cellulose also interacts favorably with the PVA matrix, ensuring uniform coating and structural stability. Additionally, the choice of solvent (e.g., water or ethanol) during nanoparticle dispersion and composite casting has a significant influence on the final microstructure, affecting nanoparticle distribution and surface wettability.

Wettability, typically measured by contact angle analysis, is a critical surface property that governs fluid absorption, bacterial adhesion, and the formation of biofilms. It can be tuned by varying the PVA-to-nanoparticle ratio, the nature of the cellulose substrate, and the casting solvent. These variables influence surface energy, porosity, and morphology, thereby dictating how the composite interacts with biological fluids.

The antibacterial efficacy of these composites is primarily due to AgNPs; the spatial distribution and long-term stability of the nanoparticles, along with potential synergistic effects with MG, also contribute to sustaining the antimicrobial action. These features are crucial for applications such as wound dressings, hygiene products, and medical coatings where infection control is essential.

Hybrid nanostructures that combine the antimicrobial or catalytic effects of silver with the magnetic or adsorptive properties of MG are being widely explored for use in biomedical, environmental, and catalytic applications—including antimicrobial agents, drug delivery systems, wastewater treatment, MRI contrast agents, and redox or photocatalytic reactions [7,8,9,10,11,12,13,14,15]. Recent studies have demonstrated the versatility of such hybrid composites for use in active packaging, antimicrobial coatings, and hydrogel systems. For instance, Errokh A. et al. [16] developed cellulose nanofibril-based films functionalized with AgNPs, offering an eco-friendly alternative for active packaging. Bag S.S. et al. [17] proposed a PVA–gelatin hydrogel embedded with AgNPs for wound healing, emphasizing its biocompatibility and antibacterial performance. Similarly, Song S. et al. [18] introduced a bacterial cellulose–PVA–AgNP cryogel designed for wound care, notable for its absorbency, flexibility, and long-term antimicrobial activity.

Unlike conventional hydrogels or coatings, cryogelation offers distinct advantages: it uses physical rather than chemical crosslinking, eliminating toxic reagents and reducing regulatory concerns for biomedical use. The physically crosslinked network can be reversible, allowing for reprocessing or reuse. The ice crystals formed during freezing act as porogens, creating a highly porous structure ideal for biomedical, filtration, or food [2,19].

The solvent type plays a crucial role in determining the cryogel’s microstructure. The water crystallization during freeze–thaw cycles drives the gel’s pore architecture [20,21,22]. The studies also highlight the influence of mixed solvents—particularly ethanol as a co-solvent—on cryogel morphology [23,24].

While AgNPs possess potent antibacterial properties, MG particles exhibit milder antimicrobial effects [25]. In our study, we combined AgPs and MG to achieve a synergistic antimicrobial effect. Their combination is expected to provide sustained antimicrobial activity through synergism. Additionally, the electrostatic interactions or the adsorption of AgPs on the MG surface may enhance the stability and prevent their aggregation, complementing the role of capping agents in ensuring nanoparticle dispersion.

The primary aim of this research is to develop and characterize PVA-based cryogel composites embedded with AgPs and MG particles, reinforced with cellulose fibers, and to investigate how processing variables (e.g., solvent type, freeze–thaw conditions) influence their surface characteristics and biological functionality. A secondary objective is to evaluate the efficacy of different added capping agents for plant-derived synthesis of AgPs. This study introduces a novel composite that combines the mechanical flexibility and hydrophilicity of cellulose, the gel-forming ability of PVA, and the multifunctionality of metal nanoparticles.

## 2. Materials and Methods

### 2.1. Materials

The mature *Salvia officinalis* plants originated from the N-E region of Romania. Silver nitrate was supplied by S.C. Chimopar Trading S.R.L. (Bucharest, Romania). Dextrose, poly(ethylene glycol) PEG 600, magnetite Fe_3_O_4_, sodium bicarbonate NaHCO_3_, and ammonium bicarbonate (NH_4_)HCO_3_ were purchased from Sigma Aldrich and used as received. Ethanol from Silal Trading S.R.L. (Bucharest, Romania) and deionized and filtered water (Milli-Q Academic, Millipore, France) were solvents for solution preparation. The medical gauze was produced by S.C. Interagent S.R.L. (Bucharest, Romania), and the cellulose paper sheets with narrow pores of ~10 μm were from Sartorius AG (Göttingen, Germany).

### 2.2. Synthesis of Silver Particles (AgPs)

The bio-inspired synthesis of silver particles was conducted following a previously published protocol [26], with minor modifications. Dried *Salvia officinalis* leaves were ground using an electric grinder, and 25 g of the resulting powder was soaked in 250 mL of a 1:1 (*v*/*v*) water–ethanol mixture. The mixture was stored overnight in a refrigerator to allow the extraction of the bioactive compounds.

The plant extract was then processed using a rotary evaporator, initially heated for 30 min at 80 °C, followed by an additional 30 min at 60 °C, with continuous rotation at 50 rpm in a water bath. After processing, the extract was cooled to room temperature, diluted to 250 mL with the same solvent mixture, filtered through standard filter paper, and stored in a refrigerator until further use.

To adjust the pH of the extract to alkaline conditions (from pH 5 to 9), an aqueous solution of 0.1 M NaHCO_3_ was employed. For nanoparticle synthesis, 100 mL of the plant extract and 20 mL of a particle-stabilizing solution (either 0.25% *w*/*v* dextrose or 0.15% *w*/*v* polyethylene glycol, PEG) were gradually and alternately added to 100 mL of freshly prepared 0.1 M AgNO_3_ solution, using the same solvent mixture. The reaction mixture was maintained on a hotplate with magnetic stirring at 26 °C for approximately 20 h to ensure complete reduction of Ag^+^ ions by the phytochemicals.

The resulting colloidal solutions were subjected to successive centrifugation at 10,000 rpm. The supernatant was decanted, and the precipitated particles were redispersed in the solvent. The final sediments were dried under vacuum at 60 °C. Two types of silver particles were obtained based on the stabilizing agent used: **A-type AgPs**, stabilized with dextrose, and **B-type AgPs**, stabilized with PEG.

Previous studies have confirmed that *Salvia* species are rich in diverse phytochemicals, with well-characterized extract profiles [5]. In addition to terpenoids, flavonoids, and anthocyanins, compounds such as phenolic acids and hydroxycinnamic acids—recognized for their strong antioxidant activity—can effectively reduce Ag^+^ to Ag^0^ by serving as electron donors during the bioreduction process. Moreover, certain phytochemicals function as capping and stabilizing agents, enhancing nanoparticle stability and preventing aggregation [3,4,27,28,29,30].

### 2.3. Preparation of Particle Mixtures and Design of Cellulose-Based Composites

Solutions with initial concentrations of 0.1% (*w*/*v*) for the inorganic particles and 4% (*w*/*v*) for the polymer (PVA) were prepared and combined in a weight ratio of 1: 1: 100 for Ag:MG:PVA. Water or ethanol was used as the dispersion medium for the inorganic particles; however, only water was used to dissolve PVA due to its superior solubilizing capability.

Initially, the inorganic particles (AgPs and MG) were dispersed separately by sonication for 2 h in either water or ethanol using an ultrasonic bath with 42 KHz frequency and 100 W power output (Cole-Parmer Instrument Company, Vermon Hills, IL, USA). The pH of the MG suspension was adjusted to 9 using an aqueous solution of ammonium bicarbonate ((NH_4_)HCO_3_). Subsequently, the silver particle suspension was gradually added to the MG solution and stirred at 35 °C for 1 h to promote interaction and partial homogenization.

On the following day, the mixed inorganic suspension, re-homogenized by ultrasonication for 30 min, was slowly added dropwise into the PVA solution. The resulting mixture was continuously stirred for over 30 min at 35 °C using magnetic agitation to ensure uniform dispersion.

As described in Section 2.1, medical gauze and laboratory-grade filter paper were used as cellulose fiber substrates. The final composite formulation—containing PVA and both inorganic particles—was cast onto the cellulose supports placed in molds. To enhance porosity within the PVA matrix, the composites underwent freeze-drying via three 1 h freeze–thaw cycles, followed by a prolonged 18 h cycle. The materials were then dried at room temperature for one week and finally oven-dried at 40 °C for 2 h to ensure complete moisture removal.

A total of eight composite samples were prepared, starting from two types of silver particles, one type of magnetite, two types of solvents, and two types of cellulose supports (Table 1). The composites made with gauze were labeled **CPZ 1-4**, while those using filter paper were labeled **CZH 1-4**. The control samples without inorganic particles were also prepared and labeled **CPZ-M** and **CZH-M** for gauze- and paper-based supports, respectively.

### 2.4. Characterization Methods of Particles and Composites

#### 2.4.1. Transmission Electron Microscopy (TEM)

Transmission electron microscopy was used to evaluate the size and morphology of the AgPs produced via plant-mediated synthesis. The analyses were performed using a Hitachi HT7700 series TEM (Hitachi, Ltd., Minato-ku, Tokyo, Japan) operated at a maximum acceleration voltage of 120 kV. This instrument features a special design that enables imaging in “high-contrast” or “high-resolution” modes without the need for additional adjustments, allowing real-time visual inspection under daylight conditions directly on the display. The samples were prepared by depositing the nanoparticles from the liquid phase onto Ted Pella 300-mesh copper grids coated with a carbon film. The grids were then dried under conditions optimized for the specific material to minimize alterations to their morphology and appearance.

#### 2.4.2. Scanning Electron Microscopy (SEM) and Energy-Dispersive X-Ray (EDX) Spectroscopy

The sample’s particle shape and chemical composition were examined using a Verios G4 UC Scanning electron microscope (Thermo Scientific, Brno, Czech Republic) fitted with an energy-dispersive X-ray spectroscopy analyzer (Octane Elect Super SDD detector, Pleasanton, CA, USA). For SEM analysis, the samples were mounted on aluminum stubs using double-sided carbon adhesive tape and coated with a 4 nm layer of platinum with a Leica EM ACE200 sputter coater (Vienna, Austria) to ensure electrical conductivity and minimize charge accumulation under the electron beam. The morphological characterization was performed with a secondary electron detector-Through Lens Detector (TLD) in high-resolution mode at an accelerating voltage of 5 kV and a beam current of 0.2 nA. For EDX measurements, the samples were examined at an accelerating voltage of 20 kV and a beam current of 6.4 nA.

#### 2.4.3. UV-Vis Spectroscopy

UV–Visible spectra were recorded using a SPECORD 200 spectrophotometer (Analytik Jena GmbH & Co., Jena, Germany) with 1 × 1 cm^2^ quartz cuvettes. Absorbance measurements were conducted over a range of 200–800 nm. The samples were prepared in either aqueous or alcoholic solutions.

Unlike bulk metals, the metallic nanoparticles acquire optical resonance during the interaction with electromagnetic waves. The collective oscillations of the surface free electrons in multiple directions —referred to as plasmonic modes—are readily excited. The principal plasmonic resonance modes are as follows:

- In-plane dipole mode: This mode gives rise to a strong, dominant surface plasmon resonance (SPR) peak in the UV–Vis region;
- Out-of-plane quadrupole mode: This mode results in a weaker and broader signal in the UV–Vis spectrum, often observed as a shoulder peak, and is typically associated with asymmetric nanoparticles or particle aggregation [31,32,33].

Usually, the appearance of a dark-brown color during the synthesis process of metallic nanoparticles is attributed to this SPR [34]. The position of the SPR band is strongly influenced by the size and shape of the metal nanoparticles, molecular interactions, as well as by the properties of the solvent. For spherical nanoparticles, an increase in the particle size generally results in a redshift of the SPR band. In the case of very small nanoparticles (typically below 20 nm), an initial blueshift may be observed as the size decreases, followed by a redshift with further size reduction. So, the spherical nanoparticles can therefore exhibit both blue- and redshifts of the SPR band depending on their size regime. In contrast, anisotropic nanoparticles—such as rods, plates, and prisms—tend to exhibit SPR bands that are red-shifted due to their elongated or flattened geometries [35,36]. Additionally, changes in the refractive index, and particularly in the dielectric constant of the solvent, can also cause shifts in the SPR band, typically resulting in a blue-shift when the solvent has a lower dielectric constant [37].

#### 2.4.4. Dynamic Light Scattering (DLS)

The particle size in aqueous and ethanol solutions was measured using a Zetasizer Nano ZS (Malvern Instruments, Malvern, UK) equipped with a 633 nm He-Ne laser and backscattering detection at 173°. For the DLS experiments, the 0.1% (*w*/*v*) particle suspensions obtained after sonication were diluted to 0.03% (*w*/*v*). The reported values represent the mean of three measurements. The zeta potential of the colloidal particles was determined by electrophoretic light scattering using the same instrument and disposable folded capillary cells.

The researchers often compare the size of nanoparticles in a colloidal dispersion with the particle size obtained after solution drying [38,39,40,41]. However, measurements from DLS and TEM can differ significantly. DLS measures the hydrodynamic diameter, which includes not only the particle core but also the solvation layer (adsorbed solvent molecules) and any dynamic shell surrounding the particle (e.g., surfactants, ligands). As an intensity-weighted technique, DLS is particularly sensitive to larger particles and potential aggregation, which can skew the results. In contrast, TEM provides the real dimensions (core physical diameter) of individual particles through direct high-resolution imaging. For these reasons, DLS measurements often overestimate particle size compared to TEM results [42,43,44].

#### 2.4.5. Fourier-Transform Infrared (FTIR) Spectroscopy

FTIR spectra were collected in the transmission mode from 400 cm^−1^ to 4000 cm^−1^ at room temperature with a resolution of 4 cm^−1^ using a Bruker Vertex 70 FTIR spectrometer (Bruker, Ettlingen, Germany). The KBr pellets were prepared by mixing the sample with KBr powder. FTIR spectroscopy was used to detect the changes in vibrational modes (peak/band shifts; band intensities) of functional groups and the specific chemical bonds. The deconvolution of the FTIR signals was performed using OriginPro 2019 software (OriginLab Corporation, Northampton, MA, USA).

#### 2.4.6. Contact Angle Measurements

The wettability of the samples was established by measuring the static contact angle values using the sessile drop method at room temperature with a CAM 101 Optical Contact Angle device (KSV Instruments, Helsinki, Finland). The solvent used was MilliQ water. A Hamilton syringe was utilized to place a drop of liquid (~1 µL) on the surface of the samples, and images were captured for analysis on a computer.

#### 2.4.7. Antimicrobial Screening

Model microorganisms such as *E. coli* (ATCC 8739) and *S. aureus* (ATCC 25923) were used for preliminary antimicrobial screening of the composites. The square-shaped filter paper- or gauze-supported composites of about 1 cm^2^ were placed on Petri plates containing Muller-Hinton agar, previously inoculated with 200 µL of overnight bacterial culture adjusted to 0.5 McFarland turbidity (∼2 × 10^8^ CFU/mL) for each strain. The plates were then incubated at 37 °C for 24 h. After incubation, the zone of inhibition was measured and expressed in mm [45]. Two antibiotics, clindamycin 2 µg (CM) and amoxicillin/clavulanic acid (AMC) 30 µg (20/10), represented the positive control, while CPZ-M-H_2_O, CPZ-M-EtOH, CZH-M-H_2_O, and CZH-M-EtOH were designated as negative controls. The test was carried out in triplicate.

#### 2.4.8. Statistical Analysis

The statistical analysis was performed using OriginPro 2018 software (OriginLab Corporation, Northampton, MA, USA), and results were expressed as mean ± standard deviation (SD) of three replicates (n = 3). All data were analyzed by one-way ANOVA, followed by Student’s *t*-test (*p* < 0.05) for contact angle measurements, or Tukey’s HSD post hoc test (*p* < 0.05) for multiple comparisons of antimicrobial screening.

## 3. Results and Discussion

### 3.1. Morphology and Size of Silver Particles in Bulk

The morphology of AgNPs can vary significantly depending on the synthesis conditions. When using different *Salvia* species, previous studies have reported the formation of spherical, hexagonal, polygonal, triangular, or pyramidal nanoparticles [15].

**Figure 1:**
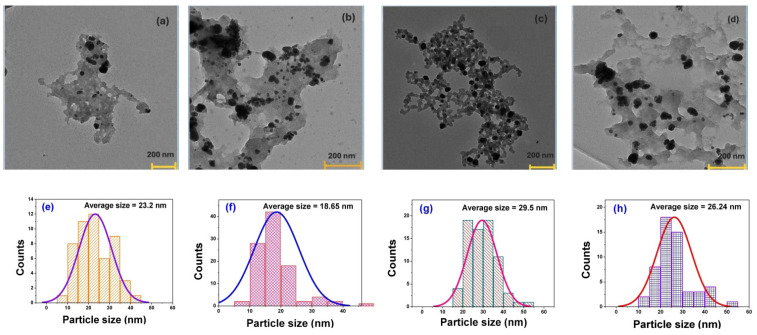
TEM micrographs (scale of 200 nm) and size distribution histograms of A-type AgPs dried from water (**a**,**e**) or ethanol (**b**,**f**), and B-type AgPs dried from water (**c**,**g**) or ethanol (**d**,**h**).

In the present study, we predominantly obtained spherical AgPs, which appeared embedded or sequestered within a matrix of organic residues—likely plant-derived compounds. Representative transmission electron microscopy (TEM) images of dry samples obtained from both aqueous and alcoholic dispersions are shown in Figure 1a–d.

To determine particle size distributions, 50–100 particles from each sample were analyzed using ImageJ software. The resulting histograms were used to calculate average particle sizes for each type of AgP. As expected, A-type AgPs, stabilized with small-molecule agents (e.g., dextrose), exhibited smaller average diameters, ranging from 18 to 23 nm. In contrast, B-type AgPs, coated with polymeric stabilizers (e.g., PEG), were larger, with sizes ranging from 26 to 30 nm (Figure 1e–h).

The choice of solvent used during particle dispersion and drying for TEM grid preparation also influenced the observed particle size. The particles dried from ethanol generally displayed smaller sizes and better separation compared to those dried from aqueous solutions. This suggests that ethanol enhances particle dispersion upon solvent evaporation, a well-documented phenomenon exploited in TEM sample preparation to improve the resolution and reduce particle agglomeration.

### 3.2. Chemical Composition of Inorganic Particles

**Figure 2:**
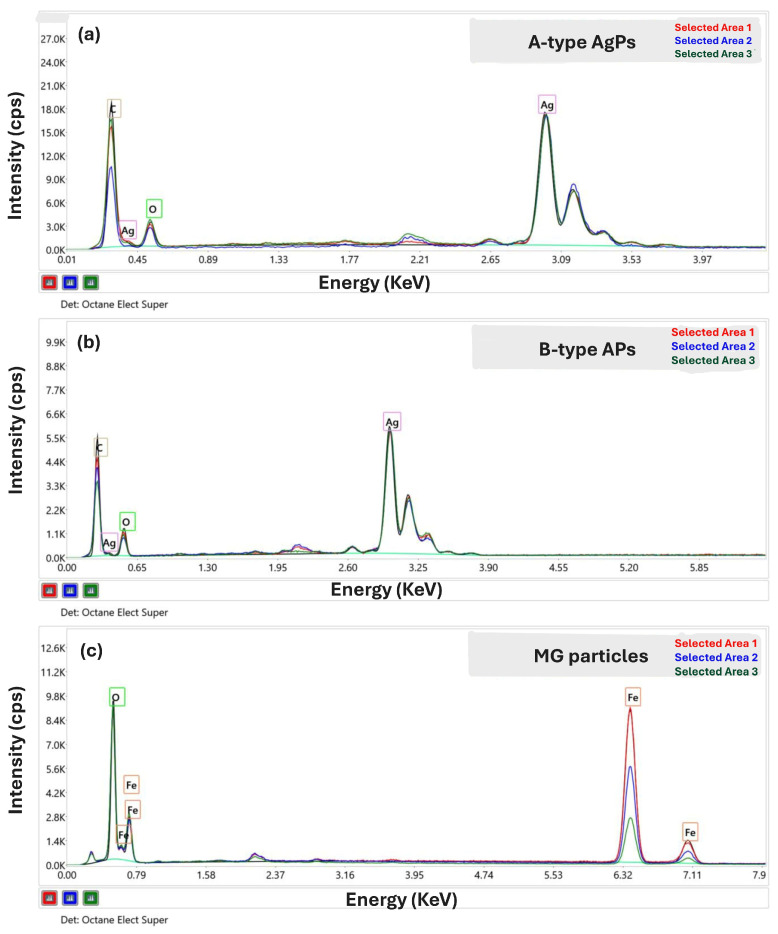
EDX Spectra of A-type AgPs (**a**), B-type AgPs (**b**), and MG particles (**c**).

EDX spectroscopy results are presented in Figure 2 and Table 2 and were used to identify and quantify the elemental composition of the inorganic particles.

For both A-type and B-type AgPs, characteristic peaks corresponding to silver were observed—most notably the intense Ag(L) peak at 2.984 keV, associated with L-shell electronic transitions. These peaks confirm the successful synthesis of AgPs in both types. Additional peaks corresponding to carbon (C(K), 0.27 keV) and oxygen (O(K), 0.52 keV) were also detected. These signals are attributed in part to residual organic compounds originating from the *Salvia officinalis* extract used during the green synthesis of AgPs [46]. These plant-derived residues are known to function similarly to capping agents, stabilizing nanoparticles during formation [12].

Similar results were reported by Sreckovic N.Z. et al. [47] using *Salvia pratensis* L. extracts, where the phenolic compounds were identified as primary contributors to nanoparticle surface functionalization. In addition, some authors have noted that ambient oxygen adsorbed onto the sample or the SEM stub may also contribute to the O(K) signal at 0.52 keV, albeit to a lesser extent [48,49].

A small platinum peak (Pt(M)) at 2.04 keV was also present in Figure 2a,b, originating from the thin platinum coating applied during SEM sample preparation. Notably, a weak signal at approximately 2.6 keV was also observed near the Pt(M) peak, corresponding to chloride. This signal supports the presence of chlorinated compounds within the plant extract used for AgPs synthesis.

The EDX spectrum of the MG sample (Figure 2c) confirmed the presence of both oxygen and iron. A dominant peak at 6.39 keV corresponds to the K-shell transition of iron, while the oxygen signal remained evident at 0.52 keV. Additionally, a discrete carbon signal (0.27 keV) was observed in the MG spectrum, suggesting the presence of residual organic compounds from the synthesis process. Similar findings were reported by Prabhu Y.T. et al. [50], who noted low-intensity carbon signals in MG nanoparticles synthesized with organic precursors.

Qualitative EDX analysis of the inorganic particles revealed the presence of silver, carbon, oxygen, iron, and trace chlorine in varying proportions (Table 2). Quantitative analysis showed that the silver content in AgPs was approximately 66 wt%, while the iron content in MG was around 81 wt%. These values represent the average composition obtained from measurements taken across three different areas of each sample surface.

### 3.3. Surface Properties of Inorganic Particles and Their Mixture

**Figure 3:**
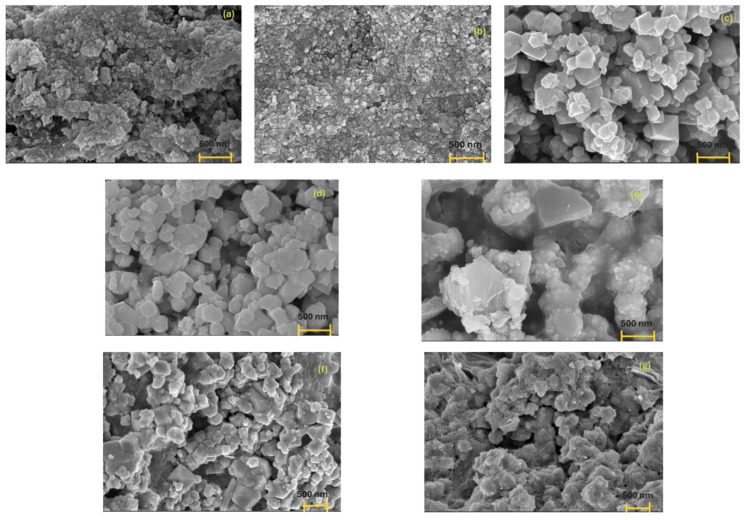
SEM micrographs (scale of 500 nm) of dried samples: A-type AgPs (**a**), B-type AgPs (**b**), MG particles (**c**) and their mixtures: MIX 1 (A-type AgPs+MG); MIX 2 (B-type AgPs+MG); MIX 3 (A-type AgPs+MG); MIX 4 (B-type AgPs+MG) (**d**–**g**).

AgPs were combined with MG in different solvents to form composite mixtures. Mixtures prepared in water were designated as MIX 1 (A-type AgPs with MG) and MIX 2 (B-type AgPs with MG), while those prepared in ethanol were labeled MIX 3 (A-type AgPs with MG) and MIX 4 (B-type AgPs with MG). The size and surface morphology of the dried individual metallic particles and their mixtures were examined using SEM analysis. The AgPs synthesized via the plant-assisted method were predominantly spherical and smaller in size compared to the polyhedral, angular particles (e.g., cubic, hexagonal) characteristic of commercial MG, as shown in Figure 3a–c.

In all inorganic particle mixtures (MIX 1–4), two distinct populations of particles with different sizes and morphologies were identified. One population consisted of smaller particles, approximately an order of magnitude smaller than the other. These smaller AgPs (Figure 3a,b) were not well-individualized, likely due to the presence of organic components such as *Salvia* sp. extract, dextrose, or PEG. In contrast, larger, unstabilized MG particles with well-defined polyhedral shapes were visible (Figure 3c). SEM images (Figure 3d–g) further revealed notable morphological differences between samples dried from water and those dried from a water–ethanol solvent mixture.

In MIX 1, the SEM micrograph shows a relatively homogeneous composite with MG as the dominant phase, composed of faceted MG particles sparsely decorated with A-type AgPs. The low number of distinct AgPs on the MG surface suggests that A-type AgPs were well incorporated or embedded within the MG matrix.

The rough surface observed in the MIX 3 sample can be attributed to three types of clusters:

(a) MG aggregates formed due to dipole–dipole magnetic interactions characteristic of such materials,
(b) A-type AgPs electrostatically adsorbed onto MG surfaces, and
(c) agglomerates of A-type AgPs embedded in the more irregular and disrupted MG surface.

In contrast, the micrograph of MIX 2 displays a distinct heterostructure morphology, indicating a different interaction mechanism between the inorganic components. Here, smaller, spherical B-type AgPs are anchored onto the surface of larger, irregular MG particles, resembling a core–shell configuration. The good dispersion and separation of B-type AgPs suggest stabilization via surface interactions with MG.

Compared to MIX 2, the MIX 4 sample appeared smoother, with more irregular clustering. This morphology likely results from partial embedding of B-type AgPs into the MG matrix during drying, implying stronger interactions between the two components.

In both MIX 2 and MIX 4, which contain B-type AgPs, PEG likely acts as a stabilizing or bridging agent between MG and AgPs. This is supported by SEM images showing MG particles decorated with smaller AgPs, indicating the presence of a polymeric interfacial layer.

Finally, MIX 3 and MIX 4 (dried from ethanol) appear more compact than their water-based counterparts (MIX 1 and MIX 2). This may be because dextrose and PEG act as more effective capping agents in aqueous environments. The solvent system influenced the final morphology of the mixtures, with enhanced stability of B-type AgPs in the MG matrix observed in ethanol-dried samples.

### 3.4. Optical Properties of Inorganic Particles in Solution

The AgNPs are also known to have a distinct SPR peak, and their absorption band is influenced by the shape and the size of the nanoparticles, too [51,52].

**Figure 4:**
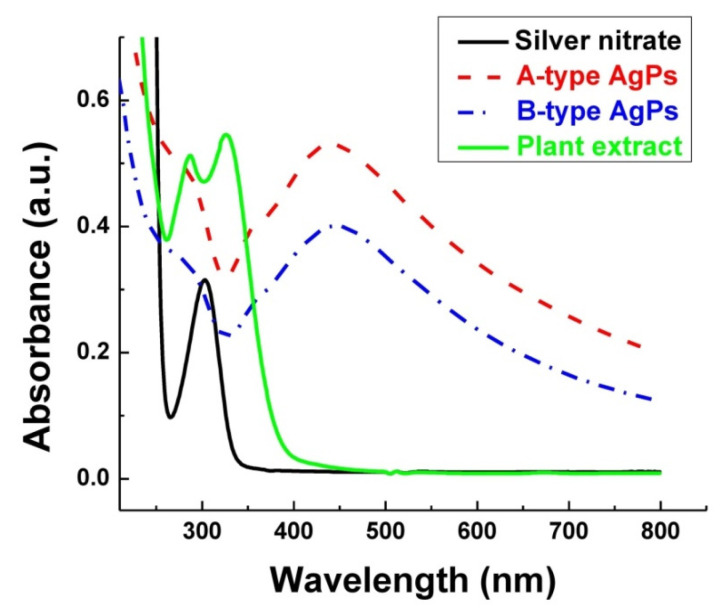
UV-Vis spectra of plant extract, silver nitrate, and AgPs.

**Figure 5:**
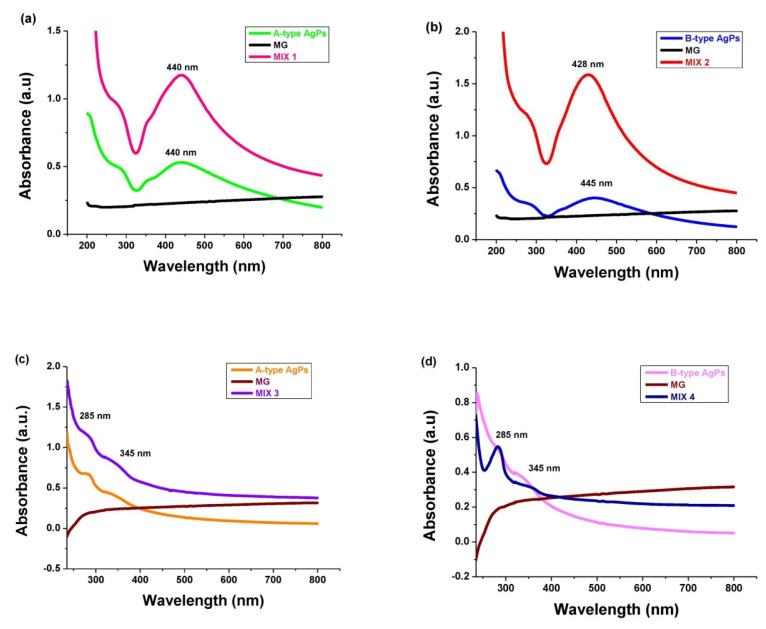
UV-Vis spectra of AgPs, MG, and their mixtures in water (**a**,**b**) and in ethanol (**c**,**d**).

The UV-Vis spectra of our AgPs obtained using *Salvia officinalis* extract and of MG particles, together with their mixtures in water and ethanol, are presented in Figure 4 and Figure 5a–d. The λ_max_ of A-type and B-type AgPs was observed at 440 nm and 445 nm, respectively. This result is consistent with other studies, confirming that the chosen synthesis method was successfully implemented, and our AgPs were spherical, with diameters smaller than 80 nm, as already evidenced by TEM analysis [52,53]. Compared to a previous study [26], the UV-Vis absorption peak, specific to each type of newly synthesized AgPs, was better defined and narrower. As Figure 4 shows, the absorption maximum peak of AgNO_3_ is not found in the spectra of the AgPs, suggesting that the precursor has been completely reduced by the phytochemicals.

Regarding the optical properties of the MG in the UV-Vis range, the results reported in the literature are contradictory. Some authors state that the MG exhibits characteristic absorption bands with peaks at 260–270 nm (Fe^2+^-O absorption band), 300–350 nm (Me-O charge transfer absorption bands involving Fe^3+^ and Fe^2+^ ions), or 420 nm (iron oxide formed by synthesis) [54,55,56]. Others report that MG absorbs mainly in the UV–Vis region due to the charge-transfer transitions between Fe^2+^ and Fe^3+^ intervalence ions, such that a broad absorption without a sharp SPR peak (usually specific to metals) was recorded [57,58]. Also in our study, MG presented no absorption characteristic peaks in the UV-Vis region, regardless of the solvent used for dispersion (Figure 5).

Generally, the optical properties of the inorganic particles in solution changed after mixing the AgPs with the MG, depending on the solvent used for their dispersion (Figure 5).

The electrostatic interactions between B-type AgPs and MG in water caused a so-called “blueshift” for λ_max_ from 445 nm to 428 nm. Also, the changes in the particle sizes after mixing could be correlated with this hypsochromic effect on the SPR peak. For A-type AgPs, no change in the position of the SPR band recorded at 440 nm after their introduction into the MG aqueous dispersion. Some studies have reported a blueshift of in-plane dipole plasmon bands of 5 nm in the case of silver nanospheres, or even of 20 nm and 40 nm for silver nanoprisms with different dimensions, when the solvent was changed [59,60].

The AgNPs should also have the plasmon absorption maximum in ethanol between 400 and 450 nm [61]. In our study, the SPR band of AgPs observed at 440 nm or 445 nm in aqueous solutions is no longer detectable in alcoholic media. The adsorption of ethanol molecules on the nanoparticle surface can further dampen electron oscillations, thereby weakening the SPR signal. Additionally, this apparent disappearance of the SPR peak upon solvent change is an artifact rather than an actual loss of plasmonic response. The “disappearance” of the SPR peak of AgPs in ethanol has been correlated to the reduced colloidal stability and aggregation induced by the lower polarity of the solvent; dielectric constant ε of ethanol and water are about 24 and 80, respectively [62]. Therefore, we will rely on indirect evidence from shifts in the UV–Vis spectra and monitor the shoulder of the SPR band, which appears around 355 nm in water and is more pronounced for A-type AgPs. The shoulder-like weaker broadband signal from 355 nm, observed in Figure 5a,b for AgPs dispersed in water, appears broadened, flattened, and blueshifted to 345 nm in ethanol (Figure 5c,d). Because the lower polarity of ethanol reduces the electrostatic stabilization of the particle, this solvent fails to stabilize the particles, causing them to cluster like Ag-Ag, Ag-MG, or MG-MG aggregates.

Because the polyphenols, containing chromophore benzene rings, are highly soluble in ethanol, the UV band at 285 nm (Figure 5c,d) can be assigned to these phytochemical residues solvated from the surface of the AgPs [63]. This signal becomes more intense in ethanol.

### 3.5. Size Distributions and Colloidal Stability of Inorganic Particle in Solution

**Figure 6:**
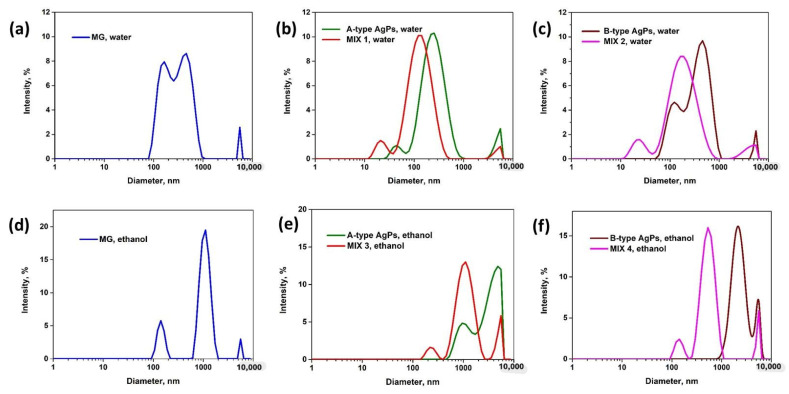
Size distribution of the MG, AgPs and their mixture in water (**a**–**c**) and in ethanol (**d**–**f**).

The particle sizes in solution, as well as their colloidal stability, were monitored through DLS and zeta potential (ZP) measurements. The Z-average diameters and ZP of the AgPs and MG and their mixture in water and ethanol are summarized in Table 3, and the size distributions are presented in Figure 6. As the principal electrokinetic property of the particles, the ZP will influence the agglomeration or dispersion phenomena in solution. It is measured in mV and evaluates the degree of electrostatic repulsion between adjacent particles [64].

The stability of magnetic particles such as MG in solution is maintained when the attractive interactions responsible for particle aggregation (van der Waals and dipole–dipole forces) are counteracted by electrostatic repulsion between charged surfaces, hydrophilic interactions between particles, or the introduction of a surface shell that provides a steric barrier [65].

MG is an amphoteric material, meaning it can act as both an acid and a base. Its surface contains Fe-OH sites that can undergo protonation (acting as Brønsted bases, meaning they accept protons (H^+^) and become Fe-OH^2+^) or deprotonation (acting as Brønsted acids), depending on the pH of the surrounding solution [66]. This amphoteric behavior is important in various applications, such as in the adsorption of molecules onto MG surfaces and in the stability of MG nanoparticles in different pH environments.

Arias J.L. and colleagues [66] analyzed the ZP–pH relationship for MG nanoparticles. They found that the isoelectric point of the MG nanoparticles dispersed in water in the presence of 10^−3^ M NaCl is around pH 7; below this pH, the ZP is positive, while above pH 7, the ZP becomes negative. The same behavior was observed by Jorge and his team [64,67].

In our study, MG particles, without a stabilizer, were poorly dispersed in water. Consequently, the inorganic particles with sizes between 100 and 1000 nm were obtained in water (Figure 6a), as shown by the SEM image, too (Figure 3c). Some large aggregates were also observed. The ZP of the MG particles was found to be −21.8 mV at pH = 9. In alkaline solution, the MG particles have a negative charge and repel other negatively charged particles. 

In the case of AgPs, the DLS measured the size of the particles coated with the organic layer (plant extract and capping agent). Using dextrose or PEG 6000 as capping agents for the synthesis of the AgPs, the colloidal stability was slightly improved compared with the old study (about 19 mV) [26]. The A-type AgPs stabilized with dextrose (Figure 6b) have a trimodal distribution in aqueous solution: small particles around 30–50 nm, larger particles around 300 nm, and also aggregates (5 μm). The B-type AgPs (Figure 6c) were covered with phytochemicals and polymer (PEG), so these particles in water also have a trimodal distribution, but with larger sizes compared with A-type AgPs. In the literature, there are also AgNPs obtained in the presence of plant extract that have bimodal or trimodal size distribution by intensity [68,69,70].

An unexpected result was when AgPs were mixed with MG (MIX 1 and MIX 2); the size distribution showed the presence of smaller particles (Figure 6b,c). A possible explanation could be that a part of the capping agent or the hydrophobic components from the plant extract switch from the surface of AgPs to MG. Thus, the nanoparticles with diameters between 10 and 40 nm observed by DLS in solution were most probably the bare AgPs, whose size coincides with the one observed by TEM (Section 3.1). The ZP of the AgPs and their mixture with MG were negative (Table 3) due to the deprotonation of the organic components at basic pH. The values of the ZP were higher than 20 mV, showing the stability of these particles in aqueous solution.

The MG particles have an even worse dispersion ability in ethanol compared with water, so the polydispersity index was very high (0.93), and particles with 140 nm, 1100 nm, and 5600 nm were observed in the size distribution by intensity (Figure 6d). The ZP of MG particles has a positive value (+25.4 mV). In ethanol (or other non-aqueous solvents), the situation is quite different. Because ethanol is a much weaker proton donor/acceptor than water, the surface reactions often involve different mechanisms, like adsorption of molecular species or ion exchange, rather than simple proton transfer. So, the Fe-OH surface sites of MG are less susceptible to protonation/deprotonation. In addition, the organic surfactants or capping agents (e.g., amines, quaternary ammonium compounds) probably used in MG synthesis and adsorbed onto the particle surface could be a possible cause of positive ZP for MG in ethanol. These agents may remain protonated in alcohol and contribute to the net positive surface charge of MG. So, the positive ZP of MG is mostly due to the balance between surface functional groups and the limited ionization or counter-ion screening in ethanol compared to water. In other words, a positive ZP of MG may result from incomplete surface charge compensation.

The AgPs, covered with capping agents and plant extract, have higher diameters in ethanol compared with water (Table 3 and Figure 6). This can be because dextrose and PEG do not work as stabilizing agents in non-aqueous systems, as can be seen from the small values of the ZP in ethanol (Table 3), so the AgPs have a higher tendency to aggregate in ethanol, compared with water. After mixing the AgPs with MG, the size of the particles decreases. This can be due to the interaction of the ethanol-soluble compounds from the plant extract with the MG particles. The surface of the MG would become negative, explaining the ZP values of the MIX 3 and MIX 4.

Silver, being a diamagnetic material, diminished the direct magnetic interactions between MG particles, thereby reducing their tendency to agglomerate. Consequently, coating MG particles with AgPs enhanced their colloidal stability, particularly in aqueous environments, as other studies confirmed [71].

### 3.6. Interactions and Functional Groups of Inorganic Particles

FTIR spectroscopy is widely applied for identifying and characterizing inorganic particles. In many studies on plant-based synthesized AgNPs, the discussion often focuses only on bands from phytochemicals—such as those at 3400–3200 cm^−1^ (–O–H stretch), 3000–2900 cm^−1^ (–C–H stretch), and 1750–1600 cm^−1^ (–C=O stretch)—without addressing the fingerprint region (1000–400 cm^−1^) characteristic of the metallic component itself [8,10,11,12,13,14,58,72].

Our FTIR investigation (Figures S1–S4—Supplementary Materials) examines common peaks from organic components used in particle synthesis (AgPs or MG), and distinct peaks in the fingerprint region specific to metallic particles—whether bare or in mixtures.

(1) The signals centered at ~3435 cm^−1^, from 3670 to 3070 cm^−1^ broad strong region, are assigned to the stretching vibration of hydroxyl (−OH) or H−OH, and N−H, which could be arising from alcohols, phenols, carboxylic acids, amines, or amides. Remarkably, this band is broader in ethanol-dried samples—especially AgPs—due to enhanced hydrogen bonding.
(2) The 3010–2800 cm^−1^ band with a maximum at ~2920 cm^−1^ is common to the alkanes possessing CH_2_ or CH_3_ groups and is associated with the aliphatic C-H stretching vibration of sp^3^ carbons.
(3) The 1633 cm^−1^ peak in the 1760–1490 cm^−1^ region is due to the C=C stretching of alkenes, aromatics, or N–H bending (amide II) vibrations of proteins, peptides of primary amines. Combined 3435 cm^−1^ and 1633 cm^−1^ peaks may indicate moisture or surface oxidation.
(4) The 1460 cm^−1^ peak in the 1490–1230 cm^−1^ region is likely assigned to the C–N stretching or COO^−^ symmetric stretch from amine or carboxylate groups, and to the bending vibration of the C-H bond. The maximum of about 1380 cm^−1^ could be the signal of NO_3_^−^ symmetric stretch if AgNO_3_ was used for AgPs synthesis, or the vibration of the CH_2_ groups. The peak of 1260/1270 cm^−1^ was generated by the stretching vibration of hydroxyl (−OH) groups.
(5) In 1230–900 cm^−1^ region the following peaks were remarked: 1086/1096 cm^−1^ for stretching vibration of (C−O−H); 1022/1028 cm^−1^ for vibration of etheric bond (−C−O−); 998/1000/1005 only for B-type AgPs and MG dried from water, and due to the C–O–C, C–N, or C–OH stretching from alcohols, ethers, or polysaccharides.

So, the signals from the 1500–1000 cm^−1^ region were probably derived from the C–O, C–H, or COO– groups of the organic residues or coatings.

(6) In the 900–650 cm^−1^ region, the C–H out-of-plane bending of alkenes or aromatics is recorded. This region is partially overlapped with the fingerprint region of 800–350 cm^−1^ specific to the metallic particles (Figures S1–S4 and Table 4).

(1) The peak at ~840 cm^−1^, observed in the spectra of AgPs and MG particles dried from water, disappears in the MIX 1 and MIX 2 mixtures. Notably, this peak is absent in all samples dried from ethanol.
(2) The peak observed at 803, 805, and 808 cm^−1^ in the spectra of MG, A-type AgPs, and B-type AgPs dried from water slightly shifts to 799 cm^−1^ in MIX 1 (indicative of a possible blueshift) and to 801 cm^−1^ in MIX 2. These signals are attributed to the stretching vibrations of Me–N bonds and appear more intense in both MIX 1 and MIX 2.
(3) For the samples dried from ethanol, the signal observed at 804, 793, and 795 cm^−1^ for MG, A-type AgPs, and B-type AgPs, respectively, appears at 802 cm^−1^ in MIX 3 and MIX 4. These bands are also attributed to the stretching vibrations of Me–N bonds.
(4) The fingerprint of nanoparticles or bulk MG includes a main peak ~570 cm^−1^, assigned to Fe-O stretching vibration of tetrahedral Fe^3+^, and a secondary peak near 450 cm^−1^, assigned to Fe-O bending/stretching of octahedral Fe^2+^/Fe^3+^. In our study, MG showed two peaks at 562 cm^−1^ and 452 cm^−1^ for the sample dried from ethanol, and distinct peaks at 573 cm^−1^ and 451 cm^−1^ for the sample dried from water. Similar results were observed by Khalkhali M. et al. [8] at 577 cm^−1^, by Yang R. et al. [58] at 582.47 cm^−1^, and by Gharehaghaji N. and Divband B. [13] at wavenumbers of 568 cm^−1^ (in the tetrahedral position) and 430 cm^−1^ (in the octahedral position). The vibrational absorption at 573 cm^−1^ or 562 cm^−1^, corresponding to the Fe-O bond, shifted in MIX 1 (at ~556 cm^−1^), MIX 2 (at ~562 cm^−1^) and MIX 3 (at ~575 cm^−1^), indicating the successful adsorption of AgPs onto the MG surface. Similar observations were reported by Yang R. et al. [58] for their magnetic Fe_3_O_4_-loaded silver nanocomposites. In MIX 4, the specific band of MG disappeared.
(5) The AgPs spectra contain signals specific to Ag-Cl bonds: at ~569 cm^−1^, ~620 cm^−1^ and ~636 cm^−1^ for samples dried from water, and at ~623 cm^−1^ and at ~653 cm^−1^ for samples dried from ethanol. The origin of the FTIR peak attributed to AgCl compounds or Ag–Cl chemical bonds in the fingerprint spectra of AgPs synthesized using *Salvia officinalis* extracts is also discussed. Hydroalcoholic sage extracts are rich in phenolic compounds (e.g., rosmarinic acid, caffeic acid), flavonoids (e.g., luteolin, apigenin), essential oils (e.g., thujone, camphor), tannins, and terpenoids. Although detailed analyses of chloride content in these hydroalcoholic extracts are limited, Cl^−^ ions likely originate from the plant’s absorption of inorganic salts such as NaCl, KCl, or CaCl_2_ present in the soil [73,74,75,76,77]. The presence of chlorine in the synthesized AgPs is further confirmed by EDX spectra, showing a peak near 2.6 keV (Section 3.2), which can be attributed solely to the phytochemical composition rather than residual water, as Millipore-filtered water was used throughout the experiments.
(6) For the samples dried from water, the signals at 451, 457, and 457 cm^−1^ for MG, A-type AgPs, and B-type AgPs, respectively, shift to 453 cm^−1^ in the MIX 1 and MIX 2 mixtures. These peaks are attributed to the Fe-O stretching vibrations of octahedral Fe^2+^/Fe^3+^ ions, as well as to Ag–O stretching vibrations associated with AgO and AgOH.
(7) MG dried from ethanol exhibits a characteristic peak at 452 cm^−1^, while A-type AgPs and B-type AgPs show a distinct signal around 432 cm^−1^. In the mixtures MIX 3 and MIX 4, a single peak centered at 450 cm^−1^ is observed, assigned to the stretching vibrations of Fe-O and Ag-O bonds, originating from iron oxides, AgO, and AgOH.
(8) The signal at ~395 cm^−1^ appears predominantly in samples dried from ethanol and is attributed to the stretching vibrations of Fe-O and Ag-O bonds, assigned to MG and AgPs.
(9) In conclusion, the weak, indirect signatures observed in the ~800–300 cm^−1^ region serve as a distinctive fingerprint of inorganic metal particles, arising primarily from Me–O bonds and interactions, with additional contributions from Me–N and Me–Cl bonding environments.
(10) In mixtures, most of the bands observed in the deconvoluted signals come from AgPs and/or MG, but new bands were recorded at ~723 cm^−1^, ~690 cm^−1^ and ~584 cm^−1^ for MIX 1 or MIX 2, or at ~701 cm^−1^ for MIX 3, too. These new bands are due to electrostatic interactions between the two types of inorganic particles.

### 3.7. Surface Properties of Composites

**Figure 7:**
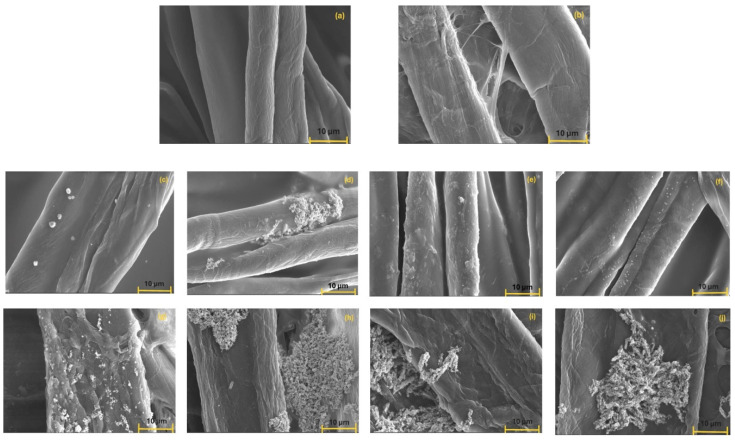
SEM micrographs (scale of 10 μm) of CPZ-M (**a**), CZH-M (**b**), CPZ 1-4 (**c**–**f**) and CZH 1-4 (**g**–**j**) composites.

The microstructure of the PVA-cellulose composites decorated with inorganic particles was influenced by the degree of compaction or volume density of the reinforcing agent (cellulose fibers), but also by the solvent used for cryogel formation, this being decisive for cryogel porosity. The SEM micrographs of composites are presented in Figure 7.

In control samples (CPZ-M and CZH-M) the paper cellulose fibers were thicker than those from the gauze (Figure 7a,b).

In case of CZH samples (Figure 7g–j), part of the PVA solution was deposited on the surfaces of the paper cellulose fibers, and another part succeeded in forming a void network, much more visible compared with the voids from gauze reinforced composites. The cellulose fibers in the paper were agglomerated by pressing during the manufacturing process; therefore, voids formed in the PVA matrix from the polymer solution remaining on the paper were then cryogelated.

By weaving, the cellulose fibers in the gauze were more aerated, and the PVA matrix had partially seeped/penetrated between the cellulose fibers, and the voids formed by the PVA cryogel were consequently less visible in CPZ samples (Figure 7c–f).

The thermal properties of the solvents (water or ethanol, respectively, from the water-ethanol mixture) influenced the porosity of the cryogels: thus, by adding ethanol, the freezing temperature/freezing rate of the composites formed from the water-ethanol mixture decreased. Simultaneously, the ethanol limited the formation of water crystals, resulting in smaller crystals compared to those formed by pure water. Thus, after the crystal thawing phase, the resulting pores were also smaller but more compact in the PVA cryogel prepared from the mixed solvent, compared to those in the PVA cryogel prepared from pure water.

The physical interaction between the inorganic particles and the cellulose fiber-reinforced PVA matrix can also be observed in the SEM micrographs (Figure 7).

In the case of composites with gauze cellulose fibers (Figure 7c–f), the inorganic particles previously dispersed in the solution were relatively uniformly arranged along the fibers, except for small agglomerations in the CPZ-2 sample. This configuration is explained by the increased density of H-bonds between PVA chains and cellulose surface groups, possibly due to the effect of a more uniform coating of cellulose fibers with the polymer solution.

In the case of composites containing filter paper (Figure 7g–j), the dispersion of the inorganic particles in the composites was not successful, so that large agglomerates in the form of networks could be observed on the cellulose fibers. These networks are more clearly visible in the case of mixtures with B-type AgPs. We assume that the various chemical treatments applied to the filter paper prevented the interaction of the PVA solution with the cellulose fibers; therefore, those PVA networks with holes remained trapped between the cellulose fibers.

### 3.8. Wettability of Composites

Wettability is a key factor in various applications, including composite manufacturing, treatment of wood, ceramic, cellulose, or PVC, and even biomedical applications [78,79]. To evaluate the wettability behavior of our composites, contact angle measurements were performed. The wettability of water on the composite surface can be characterized by the drop’s contact angle formed at the three-phase boundaries [80]. Therefore, the contact angle between the surface of the material and the liquid drop is the most important factor explaining the wettability behavior of the material. A contact angle higher than 90°, indicates lower wettability of the liquid on the solid surface.

The data presented in Table 5 indicate that both composite types exhibited contact angle values exceeding 90 degrees, demonstrating hydrophobic characteristics. Hydrophobic surfaces are important in biological applications because of their anti-fouling properties, which inhibit bacterial growth [81,82].

Generally, the impregnation of different materials can change their surface chemistry and roughness, which in turn affects their wettability [83]. Therefore, the ability of a liquid to spread on a solid surface is a complex interplay of surface chemistry, roughness, impregnation agent properties (e.g., viscosity), impregnation process, and material properties. Understanding these factors is crucial for tailoring the properties of impregnated materials for specific applications. In our study, the type of substrate used, the incorporation of inorganic particles (AgPs and MG) into these matrices, and their preparation method (repeated freeze-drying processes) has a significant contribution to the hydrophobicity of the surfaces. The analysis of the control samples (CPZ-M and CZH-M) in the absence of embedded nanoparticles indicated that the type of cellulose substrate has some influence on the wettability characteristics. The CPZ-M sample prepared using medical gauze displays a contact angle of 111.86 ± 1.26 degrees, exceeding the value recorded for the sample made of laboratory filter paper (CZH-M) of 91.33 ± 1.09 degrees. This difference could be attributed to variations in the surface topography of the cellulose fiber sources: laboratory filter paper exhibits a smoother morphology than medical gauze, which presents a more textured nanofibrous structure. Moreover, the interaction between the chemical groups of PVA and cellulose influences the wettability by reducing the number of available hydrogen bonds, leading to more hydrophobic surfaces. The incorporation of the inorganic particles into the matrix induces modifications of the surface topography as a result of variations in their size, shape, or concentration [84]. Thus, we assume that, by the inclusion of AgPs and MG in the matrices, the hydrophobicity of the surfaces increases up to 117.51 ± 0.14 at the sample CZH-4: this effect is due to the increased surface roughness of the composites, resulting from the coating of AgPs during the plant-mediated synthesis process, as well as from the larger and irregular morphology of MG particles. In short, these modifications implemented to the composite matrices had a synergistic effect in reducing surface wettability, ultimately leading to an increase in the hydrophobic character of the materials.

### 3.9. Preliminary Evaluation of the Antimicrobial Activity of Composites

AgNPs are among the most potent and widely studied antimicrobial agents. They exhibit broad-spectrum antibacterial activity against both Gram-positive and Gram-negative bacteria, including antibiotic-resistant strains. AgNPs are effective at very low concentrations, making them ideal for incorporation into biomaterials where long-term antimicrobial action is needed without cytotoxic effects on human cells. Their inclusion in the composite adds a biocidal functionality, crucial for wound dressings, medical textiles, and surfaces requiring hygienic protection.

MG particles are biocompatible, superparamagnetic materials with applications ranging from magnetic drug delivery to biosensing and pollutant removal. In composite systems, MG may contribute to antibacterial activity via Fenton-like reactions or synergy with AgNPs.

The mechanisms of action of metal particles on bacteria are not fully understood. Until now, researchers have proposed several mechanisms. One of the proposed mechanisms involves electrostatic interactions through attractive forces between metal particles with positive surface charge and negative charges of the bacterial surface. As a result of these interactions, bacteria are oxidized, and cell lysis occurs due to the nanomaterials’ released ions that interact with the thiol groups of proteins in the surface structure of the bacterial cell. Another frequently proposed mechanism refers to the occurrence of oxidative stress caused by ROS. Reactive oxygen species like singlet oxygen (^1^O_2_), hydroxyl radicals (–OH), superoxide radicals (O^2−^), and hydrogen peroxide (H_2_O_2_) could be produced by metal oxides, which interact with the DNA and different proteins, leading to cellular dysfunction [9,50,57].

Other factors that have been reported to influence the antibacterial activity are the bacterial species, the nanoparticle type, size, shape, and charge, the stability of the particles, their concentration, the type of capping agent used, or the surface chemistry [57,85,86,87].

Various composites comprising AgNPs combined with PVA and/or cellulose were assessed against clinically relevant microorganisms, including Gram-positive bacteria (*Enterococcus faecalis*, *Micrococcus luteus*, and *Staphylococcus aureus*) and Gram-negative bacteria such as *Escherichia coli* [16,17,18].

Regarding the MG nanoparticles, the antibacterial activity was evaluated using the well diffusion method against a range of bacteria, including Gram-positive species (*Bacillus subtilis*, *Staphylococcus aureus*, and *Staphylococcus epidermidis*) and Gram-negative species (*Escherichia coli*, *Pseudomonas aeruginosa*, *Xanthomonas* sp., and *Proteus vulgaris*) [50,88,89]. Generally, unlike AgNPs, the MG nanoparticles exhibited a mild inhibitory action [25].

**Figure 8:**
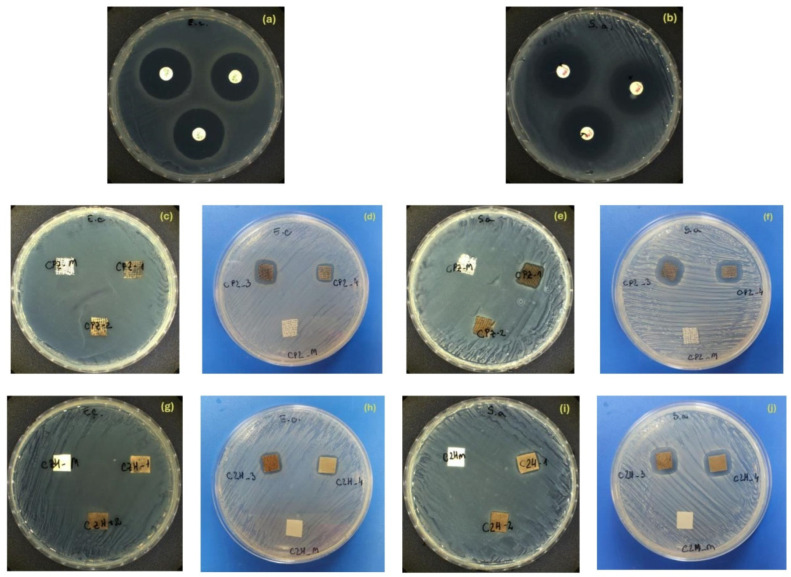
Photographs of the effect of composites against *E. coli* and *S. aureus* ((**a**)—positive control for *E. coli* (AMC), (**b**)—positive control for *S. aureus* (CM), (**c**–**f**)—Antimicrobial tests for gauze-supported composites, (**g**–**j**)—Antimicrobial tests for filter paper-supported composites).

In our study, the inhibition zones generated by the composites against the tested microorganisms are presented in Figure 8 and Table S1 (Supplementary Material). The antimicrobial potential of the composites was evaluated through a disk diffusion assay as a preliminary screening step. This method was selected to provide a comparative assessment of the tested materials and to identify the most promising candidate for further investigation.

Composites with gauze cellulose fibers (CPZ-3 and CPZ-4) and paper cellulose fibers (CZH-3 and CZH-4), respectively, exerted antibacterial activity on both bacteria, with higher inhibition zones being registered against *S. aureus* (Table S1—Supplementary Material). This difference might be related to the affinity of the composite towards the cell membrane structure of the two types of bacteria. Gram-negative bacteria like *E. coli* have an outer membrane that acts as a barrier to the entry of antimicrobial agents, limiting the access of inorganic particles from composites to the underlying cell wall and membrane, reducing their effectiveness, thus leading to smaller zones of inhibition. Unlike Gram-negative bacteria, Gram-positive bacteria such as *S. aureus* lack an outer membrane, allowing the AgPs and MG particles to bind more easily to teichoic acids and/or surface proteins, potentially causing more damage and resulting in larger zones of inhibition [90]. Moreover, considering the membrane architecture of the tested bacteria, the rigid outer membrane of Gram-negative bacteria (*E. coli*) makes them more resistant to rupture by the ROS generated by the composites, thereby increasing their resistance to metallic nanoparticles [71,91]. Similar results were also reported by Ma S. et al. [57].

CPZ-1 and CZH-1 composites inhibited only the growth of *S. aureus*, recording a zone of inhibition of 12.33 mm and 10.66 mm, respectively. CPZ-2 and CZH-2 composites were ineffective against both bacteria.

Focusing on drug release, some studies have examined how pore architecture is influenced by solvent composition. Huang X. and Brazel C.S. [92] discussed these variations, while Lozinsky V.I. [23] highlighted that water–ethanol solvent systems tend to produce less ordered microstructures with smaller pore sizes, primarily due to suppressed ice crystal formation during freezing. In our study, the antimicrobial activity was more pronounced in composites formed from water–alcohol solutions, likely due to a lower density of physical crosslinks—mediated by hydrogen bonding between the solvent and PVA—compared to purely water-based systems. The solvent mix partially inhibited freezing, but still allowed larger ice crystals than pure ethanol.

As a result, cryogels with intermediate pore size and limited connectivity were formed, restricting the deep migration of inorganic particle aggregates during synthesis. Consequently, the antimicrobial particles remained concentrated near the surface of the alcohol–water-based composites, thereby enhancing their interaction probability with bacterial cell membranes.

## 4. Conclusions

Composites based on polyvinyl alcohol (PVA) cryogels were developed by reinforcing them with cellulose fibers (from gauze or filter paper) and impregnating them with silver particles and commercial magnetite. Silver particles synthesized with *Salvia* extract exhibited size variations depending on the capping agent used (dextrose or PEG). The wettability and antibacterial performance of the composites were strongly influenced by the solvent used for dispersing the inorganic particles. Specifically, dispersions in water *versus* water–ethanol mixtures affected the freeze-drying process and, consequently, the structural and functional properties of the final materials.

Both PVA cryogelation and the incorporation of inorganic particles increased surface roughness, which enhanced the hydrophobicity of the composites. Among them, CZH-4 showed the highest hydrophobicity. Most of the composites demonstrated stronger antibacterial activity against *S. aureus*, with CPZ-3, CPZ-4, CZH-3, and CZH-4 displaying the most pronounced effects. This enhanced performance is attributed to the role of ethanol in modulating PVA matrix pore size and improving antimicrobial particle attachment and accessibility.

These findings provide a valuable first comparison among the tested composites; however, because the current assays were limited to surface-based screening, they cannot be taken as definitive potency measurements. Future studies will include quantitative analyses—such as MIC, MBC, and time–kill assays—to validate and extend these preliminary observations. To ensure safe application in medical devices (as biocides) and food-contact materials (as packaging), we will also assess cytotoxicity and particle release under physiological conditions.

Overall, this composite design represents a versatile and sustainable platform for creating multifunctional materials with tunable properties, offering promising opportunities for healthcare and advanced materials science.

## Figures and Tables

**Table 1:** Composition of PVA-cellulose composites containing inorganic particles.

Composite	Cellulose Support	Dispersant for Inorganic Particles	A-Type AgPs	B-Type AgPs	MG	PVA aq. sol.
CPZ-1	medical gauze	H_2_O	x		x	x
CPZ-2		H_2_O		x	x	x
CPZ-3		EtOH	x		x	x
CPZ-4		EtOH		x	x	x
CZH-1	filter paper	H_2_O	x		x	x
CZH-2		H_2_O		x	x	x
CZH-3		EtOH	x		x	x
CZH-4		EtOH		x	x	x

**Table 2:** Elemental Composition (weight percentage, %) at the Surface of Inorganic Particles.

	**Sample**	**A-Type AgPs**	**B-Type AgPs**	**MG**
**Elements**				
C (K)		22.03	19.28	-
O (K)		13.27	13.63	18.82
Ag (L)		64.69	67.08	-
Fe (K)		-	-	81.17

**Table 3:** DLS and ZP data for AgPs (A or B), magnetite (MG) and their mixtures (MIX) in water or ethanol.

Sample	Solvent	Z-Average Diameter (nm)	PdI	ZP (mV)
MG	H_2_O	450 ± 26	0.55 ± 0.05	−21.8 ± 0.3
A-type AgPs		224 ± 4	0.37 ± 0.01	−25.3 ± 0.7
B-type AgPs		287 ± 19	0.50 ± 0.08	−20.7 ± 0.9
MIX 1 (A-type AgPs+MG)		105 ± 0.87	0.37 ± 0.01	−28.3 ± 1.1
MIX 2 (B-type AgPs+MG)		132 ± 1.77	0.48 ± 0.30	−26.8 ± 2.3
MG	EtOH	1451 ± 110	0.93 ± 0.07	+17.8 ± 0.4
A-type AgPs		2513 ± 130	0.31 ± 0.04	−2.0 ± 0.4
B-type AgPs		3051 ± 175	0.40 ± 0.06	−1.9 ± 0.3
MIX 3 (A-type AgPs+MG)		1116 ± 107	0.40 ± 0.05	−17.2 ± 2.2
MIX 4 (B-type AgPs+MG)		782 ± 78	0.65 ± 0.01	−21.1 ± 0.4

**Table 4:** FTIR specific bands (cm^−1^) of AgPs, MG and their mixtures MIX.

MG (lit.)	AgNPs (lit.)	MG *	A-Type AgPs *	B-Type AgPs *	MIX 1 *	MIX2 *	MG **	A-Type AgPs **	B-Type AgPs **	MIX 3 **	MIX 4 **
		839	838	841							
	800–700 Ag-N st.v.	803	805	808	799	801	804	793	795	802	802
					723	723	720			723	724 723
					692	690	678			701	
	650–550 Ag-Cl st.v.	658				656		654	654	667	
			639	635							
		621	619	621	617		620	623	625	624	623
						604					
540–500 Fe-O st.v.		573			584		590			575	586
			569	569	556	562	562				
500–480 Fe-O st.v.	500–400 Ag-O st.v.	451	457	457	453	453	452			450	450
								432	432 (low)		
			417	419							420 (low)
					400	400					
		396					392		396 (low)	396	395
				381							

*Notes*: samples dried from water (*) or ethanol (**); stretching vibrations (st.v.).

**Table 5:** Contact angle values of CPZ and CZH composites and their control samples.

Sample	Contact Angle (θ^°^)
CPZ-M	111.86 ± 1.26
CPZ-1	104.75 ± 0.36
CPZ-2	112.56 ± 0.36
CPZ-3	111.40 ± 0.68
CPZ-4	115.88 ± 0.28
CZH-M	91.33 ± 1.09
CZH-1	98.42 ± 1.03
CZH-2	106.36 ± 0.99
CZH-3	116.14 ± 0.40
CZH-4	117.51 ± 0.14

*Note*: Statistical significance of 5% resulted from ANOVA variance analysis of data, followed by Student’s *t*-test, *p* < 0.005.

## Data Availability

The original contributions presented in this study are included in the article/Supplementary Material. Further inquiries can be directed to the corresponding author.

## Supplementary Materials

The following supporting information can be downloaded at https://www.mdpi.com/article/10.3390/polym17182456/s1: Figure S1: FTIR spectra of inorganic particles and their mixture MIX 1: (**a**) raw data; (**b**) fingerprint region; (**c**–**e**) signal deconvolution; Figure S2: FTIR spectra of inorganic particles and their mixture MIX 2: (**a**) raw data; (**b**) fingerprint region; (**c**–**e**) signal deconvolution; Figure S3: FTIR spectra of inorganic particles and their mixture MIX 3: (**a**) raw data; (**b**) fingerprint region; (**c**–**e**) signal deconvolution; Figure S4: FTIR spectra of inorganic particles and their mixture MIX 4: (**a**) raw data; (**b**–**c**) fingerprint region; (**d**–**e**) signal deconvolution; Table S1: The antibacterial effect of composites against *E. coli* and *S. aureus*.
