# Supplementary material for: PVA-Cellulose Fibers Composites Impregnated with Antimicrobial Particles: The Solvent Effect
Source: Polymers (Basel). 2025 Sep 10;17(18):2456. doi: 10.3390/polym17182456 (PMC12473284; doi:10.3390/polym17182456)
Supplement: Supplementary file 1 [file polymers-17-02456-s001.zip › polymers-3844779_Table S1_correction 2.pdf]

Table S1. The antibacterial effect of composites against *E. coli* and *S. aureus*.

Composite/Antibiotic	Zone of inhibition * (mm)	
	E. coli	*S. aureus*
CPZ-M-H_2O	0±0.00 ^{d}	0±0.00 ^{g}
CPZ-M-EtOH	0±0.00 ^{d}	0±0.00 ^{g}
CPZ-1	0±0.00 ^{d}	12.33±0.57 ^{ef}
CPZ-2	0±0.00 ^{d}	0±0.00 ^{g}
CPZ-3	13.33±1.54 ^{b}	18.33±1.15 ^{b}
CPZ-4	11.00±0.00 ^{c}	13.66±1.15 ^{de}
CZH-M-H_2O	0±0.00 ^{d}	0±0.00 ^{g}
CZH-M-EtOH	0±0.00 ^{d}	0±0.00 ^{g}
CZH-1	0±0.00 ^{d}	10.66±0.57 ^{f}
CZH-2	0±0.00 ^{d}	0±0.00 ^{g}
CZH-3	13.33±1.52 ^{b}	16.00±1.00 ^{c}
CZH-4	12.33±0.57 ^{bc}	15.00±0.00 ^{cd}
AMC	25.00±1.00 ^{a}	-
CM	-	27.00±0.00 ^{a}

*The values in the table represent the mean of three measurements ± the standard deviation, and include the sample sizes (~1 cm^2). Different letters within the same column indicate significant differences between samples (Tukey HSD, $p<0.05$); '-' no data available.
