# Supplementary figures and images for: PVA-Cellulose Fibers Composites Impregnated with Antimicrobial Particles: The Solvent Effect
Source: Polymers (Basel). 2025 Sep 10;17(18):2456. doi: 10.3390/polym17182456 (PMC12473284; doi:10.3390/polym17182456)

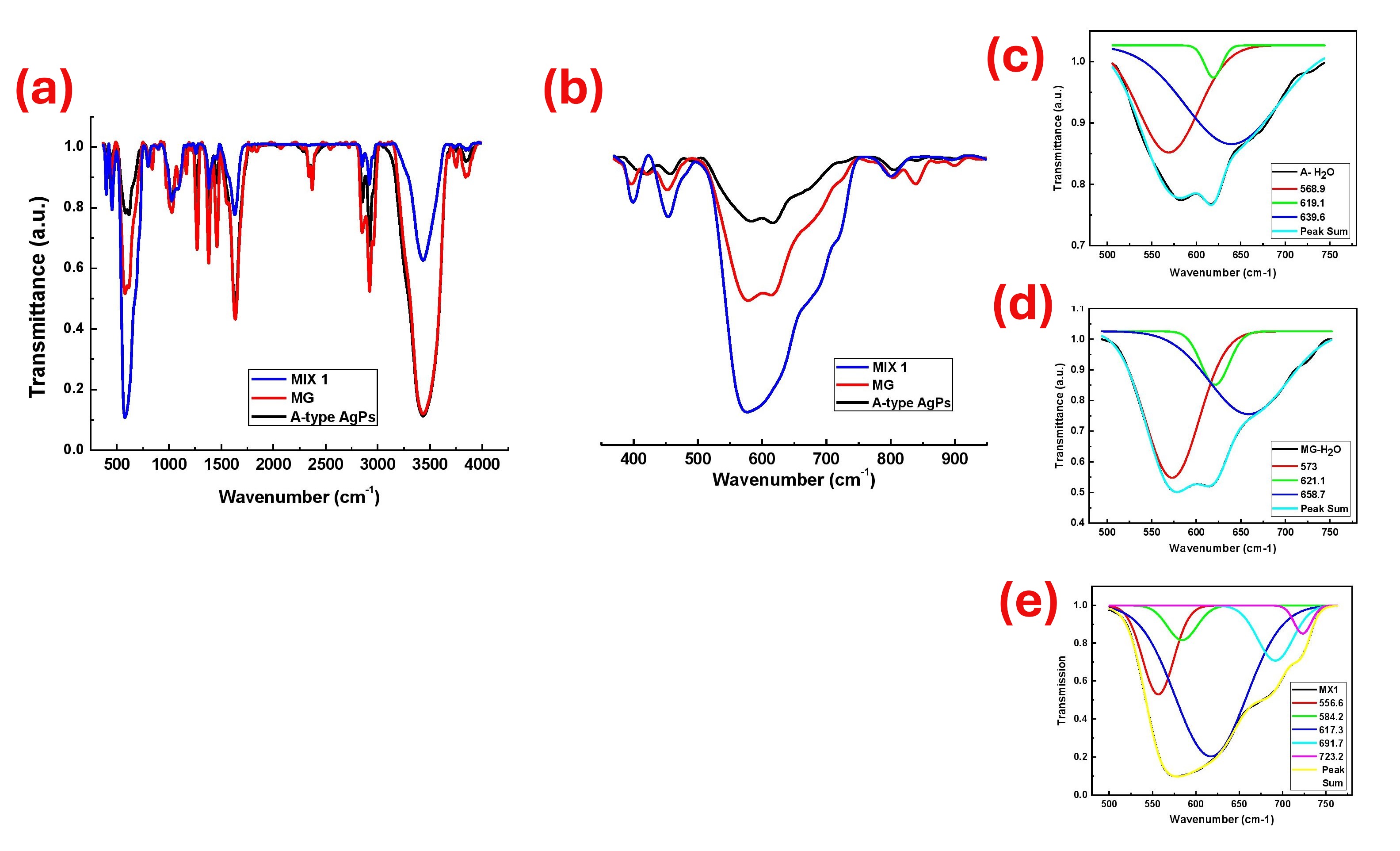

Supplement: Supplementary file 1 [file polymers-17-02456-s001.zip › polymers-3844779_figure S1_600 dpi.jpg]

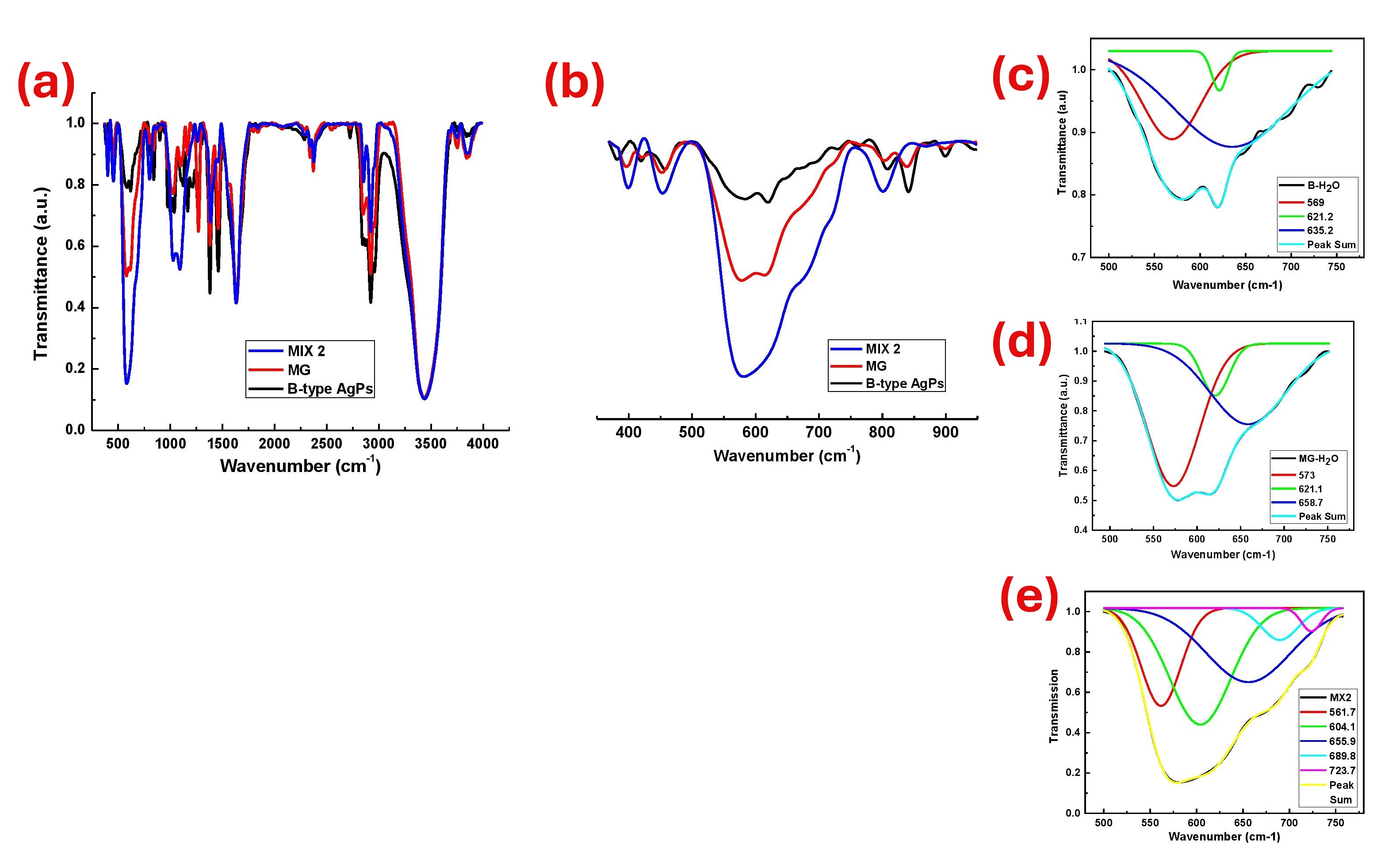

Supplement: Supplementary file 1 [file polymers-17-02456-s001.zip › polymers-3844779_figure S2_600 dpi.jpg]

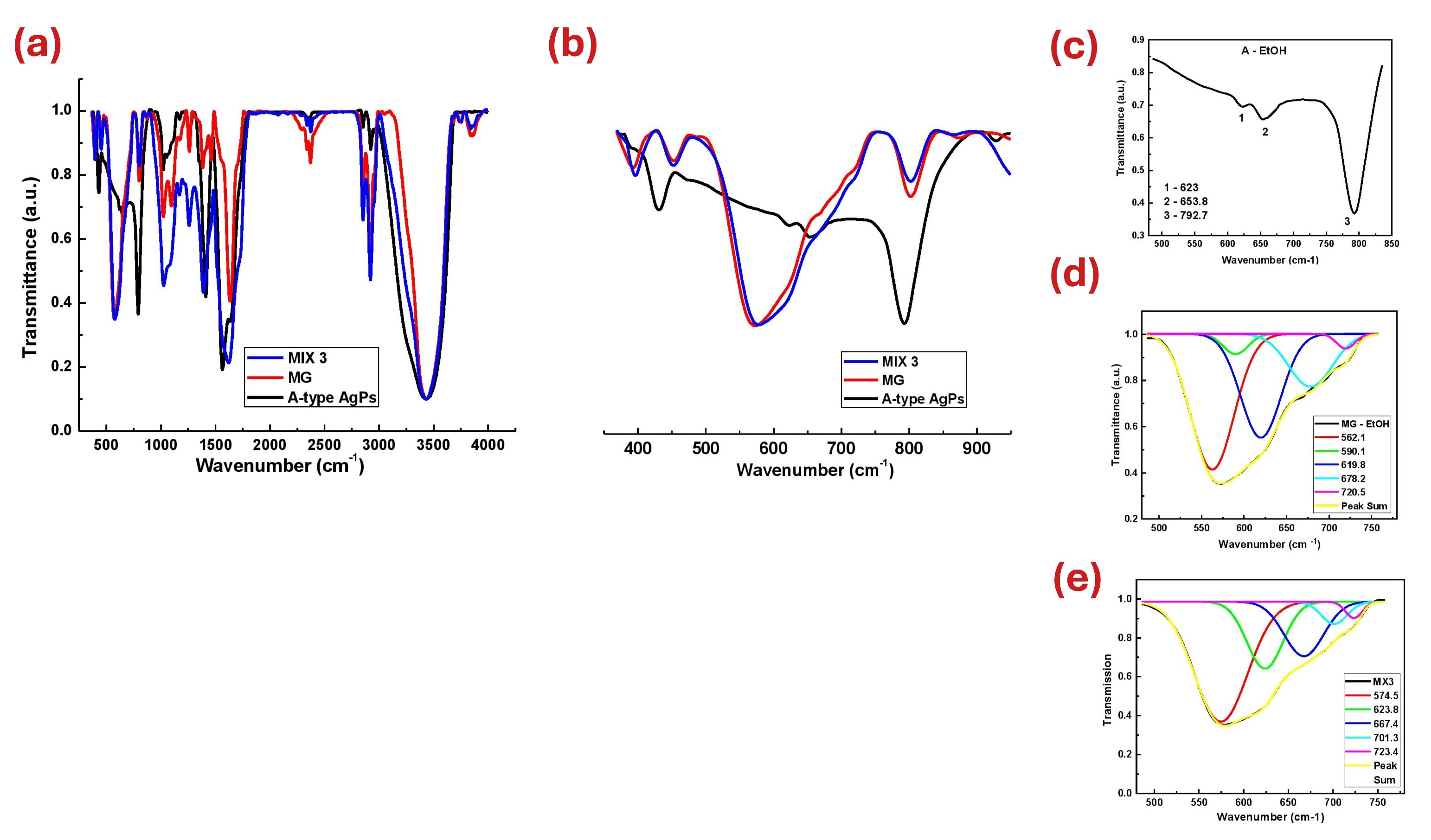

Supplement: Supplementary file 1 [file polymers-17-02456-s001.zip › polymers-3844779_figure S3_600 dpi.JPG]

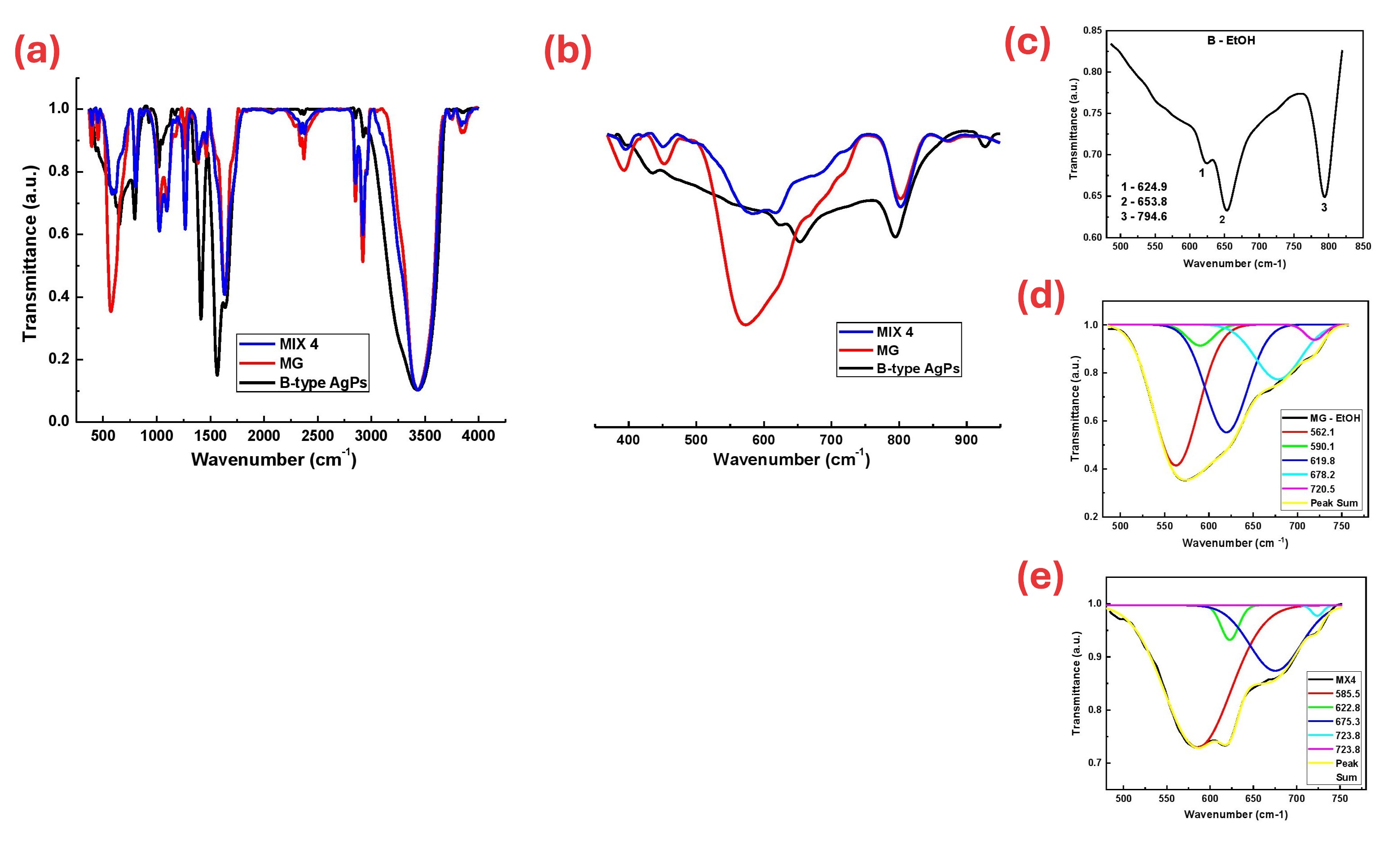

Supplement: Supplementary file 1 [file polymers-17-02456-s001.zip › polymers-3844779_figure S4_600 dpi.JPG]
